# The Cyclooxigenase-2 Inhibitor Parecoxib Prevents Epidermal Dysplasia in HPV16-Transgenic Mice: Efficacy and Safety Observations

**DOI:** 10.3390/ijms20163902

**Published:** 2019-08-10

**Authors:** Tiago Ferreira, Sandra Campos, Mónica G. Silva, Rita Ribeiro, Susana Santos, José Almeida, Maria João Pires, Rui Miguel Gil da Costa, Cláudia Córdova, António Nogueira, Maria João Neuparth, Rui Medeiros, Margarida Maria da Silva Monteiro Bastos, Isabel Gaivão, Francisco Peixoto, Maria Manuel Oliveira, Paula Alexandra Oliveira

**Affiliations:** 1 Department of Veterinary Sciences, Centre for the Research and Technology of Agro-Environmental and Biological Sciences (CITAB), University of Trás-os-Montes and Alto Douro (UTAD), 5000 Vila Real, Portugal; 2 CQVR, Chemistry Department, University of Trás-os-Montes and Alto Douro (UTAD), 5000 Vila Real, Portugal; 3 Laboratory for Process Engineering, Environment, Biotechnology and Energy, (LEPABE) Chemical Engineering Department, Faculty of Engineering, University of Porto (FEUP), 4000 Porto, Portugal; 4 Molecular Oncology and Viral Pathology Group, IPO-Porto Research Center (CI-IPOP), Portuguese Institute of Oncology of Porto (IPO-Porto), 4000 Porto, Portugal; 5 School of Health Dr. Lopes Dias, IPC, 6000 Castelo Branco, Portugal; 6 Mountain Research Centre (CIMO), IPB, 5300 Bragança, Portugal; 7 Advanced Polytechnic and University Cooperative (CESPU), Institute of Research and Advanced Training in Health Sciences and Technologies (IINFACTS), 4585 Gandra, Portugal; 8 Research Center in Physical Activity, Health and Leisure (CIAFEL), Faculty of Sports, University of Porto, 4000 Porto, Portugal; 9 Faculty of Medicine, University of Porto (FMUP), 4000 Porto, Portugal; 10 CEBIMED, Faculty of Health Sciences, Fernando Pessoa University, 4000 Porto, Portugal; 11 LPCC Research Department, Portuguese League against Cancer (NRNorte), 4000 Porto, Portugal; 12 Department of Genetics and Biotechnology and Animal and Veterinary Research Centre (CECAV), University of Trás-os-Montes and Alto Douro (UTAD), 5000 Vila Real, Portugal; 13 CQVR, Biology and Environment Department, University of Trás-os-Montes and Alto Douro (UTAD), 5000 Vila Real, Portugal

**Keywords:** COX-2, NSAID, in vivo, K14HPV16

## Abstract

Carcinogenesis induced by high-risk human papillomavirus (HPV) involves inflammatory phenomena, partially mediated by cyclooxigenase-2. In pre-clinical models of HPV-induced cancer, cyclooxygenase-2 inhibitors have shown significant efficacy, but also considerable toxicity. This study addresses the chemopreventive effect and hepatic toxicity of a specific cyclooxigensase-2 inhibitor, parecoxib, in HPV16-transgenic mice. Forty-three 20 weeks-old female mice were divided into four groups: I (HPV16^−/−^, *n* = 10, parecoxib-treated); II (HPV16^−/−^ *n* = 11, untreated); III (HPV16^+/−^, *n* = 11, parecoxib-treated) and IV (HPV16^+/−^, *n* = 11, untreated). Parecoxib (5.0 mg/kg once daily) or vehicle was administered intraperitoneally for 22 consecutive days. Skin lesions were classified histologically. Toxicological endpoints included genotoxic parameters, hepatic oxidative stress, transaminases and histology. Parecoxib completely prevented the onset of epidermal dysplasia in HPV16^+/−^ treated animals (0% versus 64% in HPV16^+/−^ untreated, *p* = 0.027). Parecoxib decreases lipid peroxidation (LPO) and superoxide dismutase (SOD) activity and increases the GSH:GSSG ratio in HPV16^+/−^ treated animals meaning that oxidative stress is lower. Parecoxib increased genotoxic stress parameters in wild-type and HPV16-transgenic mice, but didn’t modify histological or biochemical hepatic parameters. These results indicate that parecoxib has chemopreventive effects against HPV16-induced lesions while maintaining an acceptable toxicological profile in this model.

## 1. Introduction

Persistent high-risk human papillomavirus (HPV) infections are among the leading causes of cancer in the world. This infection is mainly associated with cervical cancer as well as other anogenital malignancies and a subgroup of oropharyngeal cancer [1]. Following the initial infection of the skin or keratinized mucosae, high-risk HPVs can evade the immune system and persist in their infected hosts, and may trigger carcinogenesis [2,3]. The lasting inflammatory process rises cellular levels of reactive oxygen and nitrogen species (RONS), which leads to the oxidation of proteins, lipids, and DNA [4]. Albeit HPV-induced inflammation is a primary source of ERON, HPV proteins, such as E6, E6* and E7, and even E5 indirectly, have been associated with the production and reduction of these species [5]. HPV16 and HPV18 are the most frequently detected HPV genotypes in malignant lesions [6,7,8]. HPV-induced multiphase carcinogenesis is often associated with chronic inflammation, and experimental studies in HPV transgenic mice suggest that inflammatory phenomena play an essential role in the development of intraepithelial and invasive lesions [9]. Cyclooxygenase-2 (COX-2), a key mediator of inflammation, is involved in the development of multiple types of cancer, such as colon cancer, making it a useful therapeutic target [10,11,12,13]. We have previously shown that a specific COX-2 inhibitor, celecoxib, was able to block the progression of HPV16-induced lesions in transgenic mice [14]. However, oral administration of celecoxib induced significant mortality in HPV16 transgenic mice, indicating that it has unacceptable toxicity in our animal model. In the present work, we chose a different COX-2 inhibitor, parecoxib, to achieve chemopreventive and toxicological studies in vivo using the same animal model. Parecoxib is a water-soluble prodrug of valdecoxib [15,16] and is the only selective COX-2 inhibitor that can be administered parenterally, which may contribute to a different toxicological profile compared to other COX-2 inhibitors such as celecoxib [17,18]. 

## 2. Results

### 2.1. General Results

**Figure 1:**
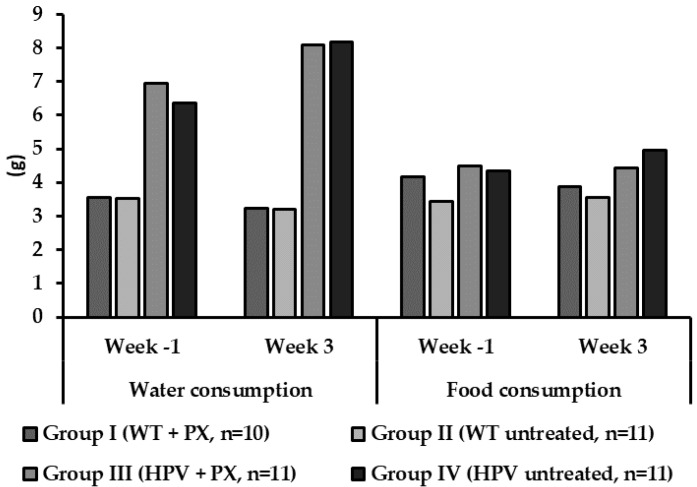
Mean values of food and water consumption per animal per day in the first and last experimental week for each experimental group. HPV—human papillomavirus; PX—parecoxib; WT—wild-type.

**Figure 2:**
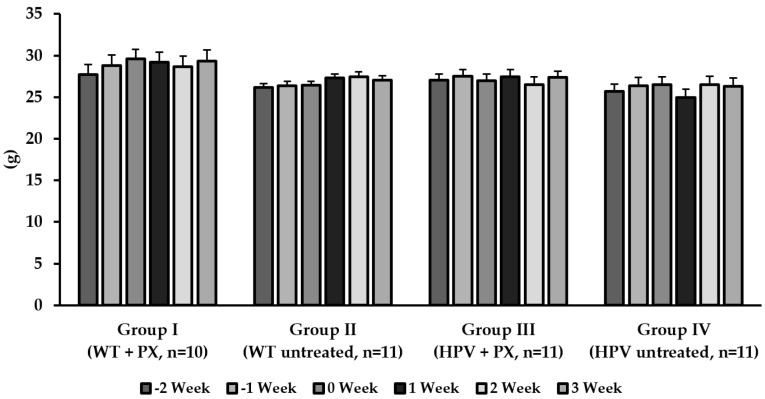
Means of the body weight of the animals at the five-week test (mean ± standard deviation). HPV—human papillomavirus; PX—parecoxib; WT—wild-type.

Humane endpoints were not reached during the study (Supplementary Table S1), and all animals survived the experimental period. Food consumption was identical between groups, but transgenic animals consumed more water than their wild-type counterparts (Figure 1). There were no significant differences concerning bodyweight between groups (Figure 2), but untreated HPV^+/*−*^ mice showed higher relative masses of the lungs, spleen, liver, and kidneys (Table 1).

### 2.2. HPV-Induced Lesions

**Figure 3:**
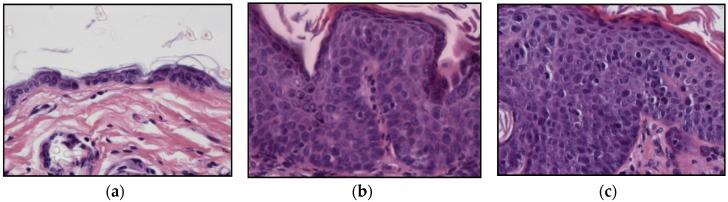
Histological lesions of skin, hematoxylin, and eosin stain. (**a**) Normal skin, 400×. (**b**) Epidermal hyperplasia, 400×. (**c**) Epidermal dysplasia, 400×.

**Figure 4:**
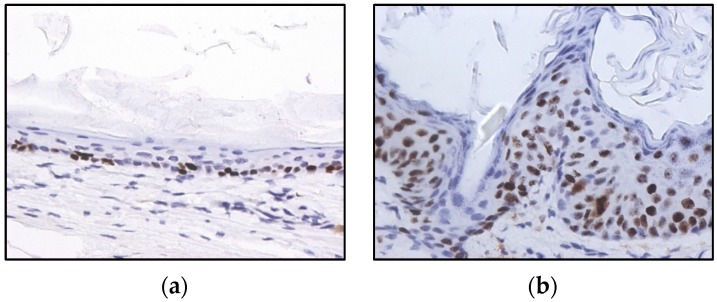
Immunohistochemistry for Ki67, in wild-type (**a**) and HPV16-transgenic (**b**) mouse skin. In panel (**a**), Ki67-positive cells are restricted to the basal epidermal layer. In panel (**b**), Ki67-positive cells are scattered through all epidermal layers, reflecting HPV16-induced aberrant proliferation. 3,3’-diaminobenzidine (DAB)–Mayer’s hematoxylin, 400×.

All ear and chest skin samples from wild type animals (groups I and II) showed normal histology (Figure 3 and Table 2). HPV16-negative animals showed a physiological distribution of proliferating epidermal cells with basal layer restricted Ki67 positive cells (Figure 4a).

**Figure 5:**
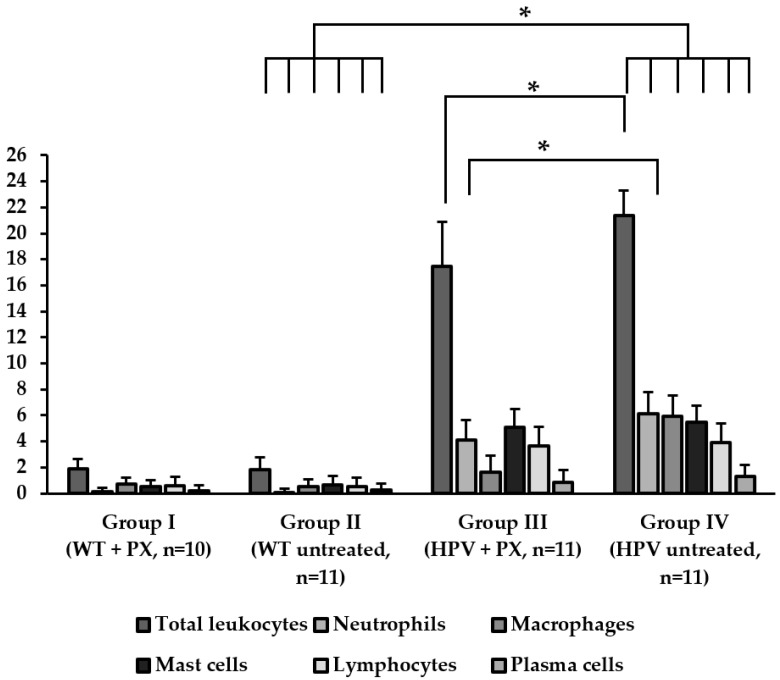
Total infiltrating leukocytes and specific leukocytic populations’ counts on healthy skin and HPV-induced lesions. Leukocyte counts were expressed as mean ± standard deviation.

All ear and chest skin samples from wild-type animals (groups I and II) showed normal histology (Figure 3 and Table 2). HPV16-negative animals showed a physiological distribution of proliferating epidermal cells, with Ki67-positive cells restricted to the basal layer (Figure 4a). On the other hand, the epidermis of HPV16-transgenic animals showed Ki67-positive cells in suprabasal layers (Figure 4b), reflecting unchecked cell proliferation, as expected. Untreated transgenic animals (group IV) showed epidermal hyperplasia (100% incidence), and 63.4% also showed multifocal to diffuse epidermal dysplasia in both the ears and the chest. Parecoxib-treated HPV^+/−^ mice (group III) showed a 100% incidence of dysplasia, but dysplastic lesions were absent in all mice (*p* = 0.027 versus untreated, group IV). HPV-transgenic mice showed increased numbers of tumor-associated leukocytes compared with wild-type animals (Figure 5). Parecoxib reduced leukocytic infiltration in HPV-induced lesions (Figure 5).

### 2.3. Hepatic Toxicity

**Figure 6:**
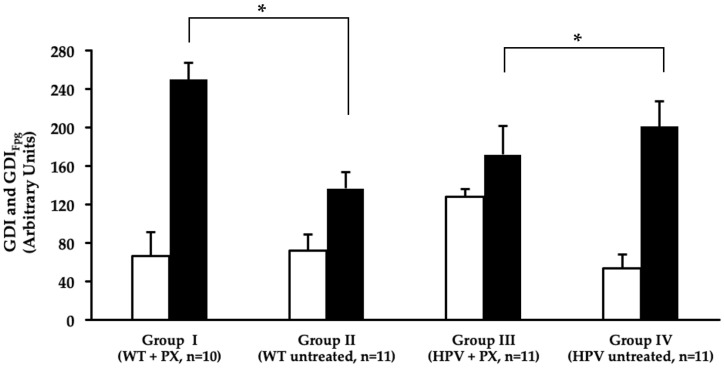
Mean values of genetic damage index (GDI), measured by the alkaline comet assay, in the liver (average ± standard deviation). Treatment without Fpg treatment (GDI) represented in white and treatment with Fpg (GDI_Fpg_) are shown in black. HPV—human papillomavirus; PX—parecoxib; WT—wild-type. ^a^—Statistically significant differences in GDI_Fpg_ versus group II (*p* < 0.05). ^b^—statistically significant differences in GDI versus group IV (*p* < 0.05).

**Figure 7:**
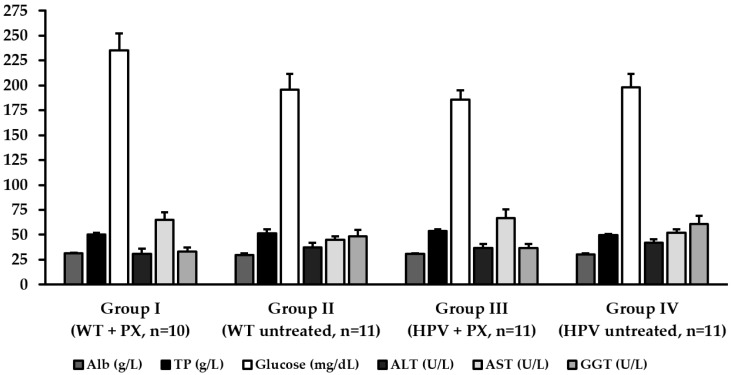
Biochemical parameters (mean ± standard deviation). Alb—albumin; ALT—alanine aminotransferase; AST—aspartate aminotransferase; GGT—gamma glutamyl transferase; HPV –human papillomavirus; PX—parecoxib; TP—total proteins; WT—wild-type.

The comet assay didn’t reveal significant genotoxic damage to the liver in association with the HPV16 transgenes. However, parecoxib significantly increased the total genetic damage index (GDI) in HPV^+/−^ mice compared with matched untreated controls. However, it slightly reduced the oxidative damage revealed by Fpg (Figure 6). Only one HPV^+/−^ mouse control (group IV) showed mild hepatitis, characterized by Kupffer cell hyperplasia and multifocal microabscesses, while mice in all other groups showed normal liver histology. In line with these findings, the plasma concentrations of albumin (Alb), total proteins (TP), glucose, alanine aminotransferase (ALT), aspartate aminotransferase (AST) and gama glutamyl transerase (GGT) were not statistically different between groups (Figure 7).

### 2.4. Oxidative Stress

The results presented in Table 3 showed that transgenic mice control group (group IV), when compared with wild-type control group (group II), have a slight increase in all enzymatic activities and lipid peroxidation, with exception made to catalase (CAT) and the GSH:GSSH ratio, but these values don’t have statistical significance. Analyzing parecoxib effects on wild-type and HPV groups (group I vs. group II and group III vs. group IV), CAT activity showed no differences, superoxide dismutase (SOD) activity was decreased in both cases but only with statistical significance between transgenic-mice groups (*p* = 0.0072), glutathione S-transferase (GST) activity had no changes and glutathione reductase (GR) had an increase in its activity, but differed only between wild-type groups (*p* = 0.0028). Lipidic peroxidation showed a statistical decrease for wild-type groups (group I vs. group II; *p* = 0.0047) and the ratio of GSH:GSSG increased in treated groups, but is only statistically different to the wild-type ones (*p* = 0.0042).

## 3. Discussion

Chronic inflammation is associated with factors like reactive species of oxygen and nitrogen, cytokines, chemokines, growth factors, and specific microRNAs, which often contribute to cancer development [19]. In most epithelial cells, COX-2 is absent or expressed at low levels but is a major contributor to inflammation and is up-regulated in many cancers, [20], so it is a relevant therapeutic target for cancer prevention and treatment [10,11,12,13]. Studies in K14-HPV16 mice have demonstrated that COX-2 is overexpressed in skin lesions induced by the HPV16 early genes [20]. Non-steroidal anti-inflammatory drugs (NSAIDs) are a class of drugs prescribed due to their analgesic, antiplatelet, and anti-inflammatory properties [21,22]. NSAIDs are useful in preventing the development of colorectal adenomas [23]. However, the existing clinical data does not support the use of COX-2 inhibitors for preventing the progression of cervical intraepithelial lesions [24]. Another difficulty is the potential toxicity of some NSAIDs, as observed with a previous study of celecoxib in K14-HPV16 mice [14]. Parecoxib, a water-soluble prodrug which is biotransformed in the liver by enzymatic hydrolysis and converted into its active metabolite, valdecoxib, is a selective COX-2 inhibitor used to minimize postoperative pain in noncardiac surgeries. It is also the only selective NSAID marketed that allows parenteral administration [17,18].

K14-HPV16 mice in an FVB/n background express all the early genes of HPV16 under the control of the human cytokeratin 14 promoter. These animals develop multi-step lesions, associated with progressive inflammation, in squamous epithelia such as the skin, similar to HPV-induced lesions in patients [25,26,27]. These animals also show severe systemic inflammation and are particularly susceptible to hepatic damage, as previously demonstrated by our group [28]. In view of the main role of oxidative stress in triggering and prolonging hepatic damage and the role of inflammation in triggering oxidative stress, we have studied some biomarkers of oxidative stress in liver samples of transgenic (HPV16^+/−^), and wild type-mice (HPV16^−/−^) treated or not with parecoxib. 

In the present study, we made two major sets of observations. The first, concerning the efficacy of parecoxib against HPV16-induced lesions, showed that this drug completely abrogated the onset of epidermal dysplasia in our animal model. Secondly, we observed that parecoxib displayed a favorable toxicological profile concerning several general and liver-specific parameters and the proper functioning of antioxidant defenses.

We observed that HPV^+/−^ mice treated with parecoxib showed only epidermal hyperplasia that did not progress to dysplasia, while 63.6% of untreated HPV^+/−^ mice showed epidermal dysplasia. This is of great importance, given that the dysplastic stage precedes the development of squamous cell carcinoma in this model, suggesting that parecoxib may able to block the development of HPV16-induced cancer. A previous study using celecoxib showed similar results [14]. Another study employing two dietary polyphenols (curcumin and rutin) with some unspecific COX-2 inhibitory activity, also blocked the development of epidermal dysplasia. In this study, they also observed a decrease of COX-2 expression and the decrease of total leukocytes and leukocyte population [20]. We found, in transgenic animals treated with parecoxib, a decrease of total leukocytes, especially of neutrophils that are involved in inflammation and are attracted to prostaglandin E2, suggesting COX-2 is inhibited by parecoxib. Celecoxib was shown to promote the activation of cytotoxic T lymphocytes present in cutaneous lesions, which may explain its activity against epidermal lesions in this model [14]. Further studies should address this topic, determining whether parecoxib exerts a similar effect over T cells and if other mechanisms are also involved. 

O’Donoghue et al. [29] employed a 10-fold lower parecoxib dose (0.5 mg/kg/day) intraperitoneally for 15 days against breast cancer mouse xenografts, and observed inhibition of tumor growth and metastasis. An even lower dose of parecoxib (0.22 mg/kg/day) administered for eight weeks has been shown to suppress the growth of esophageal adenocarcinoma xenografts in athymic mice [30]. Smakman et al. [31] employed the same dose of parecoxib as we used (5.0 mg/kg/day) for six days after sowing colon cancer cells in the liver and observed a reduced proliferation of cancer cells. In contrast, another study failed to identify antitumor effects of parecoxib at the same dose on Fischer rats with brain tumors [32]. In our study, 5.0 mg/kg/day was an effective and well-tolerated dose. We only used a single dose of parecoxib, since previous studies have indicated that it is effective and therefore, we could reduce the total number of animals sacrificed.

During the experimental protocol, the animals were monitored for signs of disease and distress that could reflect toxicity. Importantly, no changes in the behavior and physiology of the animals were observed. There were also no significant differences concerning body weight or food consumption, supporting the hypothesis that parecoxib was safe in the present experimental conditions. Transgenic animals had a higher water consumption, as previously reported for this animal model [28]. 

We also observed significant differences concerning the relative mass of internal organs between wild-type and transgenic mice, which had also been reported for these animals. Transgenic animals treated with parecoxib showed near-normal values for the relative masses of internal organs, suggesting that this compound may act systemically to re-establish homeostasis in this setting. 

Parecoxib did not induce detectable hepatotoxicity at the biochemical or histological levels. In wild-type animals, parecoxib did not alter the baseline levels of genetic damage. However, in transgenic animals, parecoxib increased the total genetic damage index (GDI) while slightly reducing the GDI_Fpg_. This is likely reflecting the hepatic biotransformation of parecoxib into valdecoxib, and suggests that DNA-repair mechanisms could cope with parecoxib-induced genetic damage, so that it didn’t translate into to biochemical or morphological changes. 

Oxidative stress is caused by an imbalance between oxidants–antioxidants that favors the oxidants, leading to the excessive generation of free radicals, particularly reactive oxygen species (ROS), and subsequently to biological damages [33]. Our results on oxidative stress parameters (GSH:GSSG and LPO) shows that the inflammation caused by HPV16 induces oxidative stress. Therefore, antioxidant system activation is required as can be seen by the increase of SOD and GR when comparing the transgenic mice without treatment (group IV) and its own control (group II). Our data shows that the inflammation caused by HPV16 induces oxidative stress, considering the increased activity of two critical enzymes of the antioxidant system (SOD and GR), which was not sufficient to prevent oxidative damage as demonstrated by the decrease of the GSH:GSSG ratio and increase of lipid peroxidation (TBARS).

Regarding the results of transgenic mice (HPV16) treated with parecoxib (group III) and its control (group IV), it seems that parecoxib leads to a decrease in oxidative stress since lipid peroxidation decreases and the GSH:GSSG ratio increases, although the latter is not statistically significant. The reduction of ROS production, and therefore the lower oxidative stress observed in groups treated with parecoxib will reduce COX-2 expression, leading to the inhibition of prostaglandin synthesis and, subsequently, proinflammatory cytokines which are also responsible for increased free radical production [34]. For oxidative stress enzymes, SOD has a significant decrease in its activity may be due to the lower generation of its substrate (O_2_^•−^), and GR, GST, and CAT didn’t show any differences. GST activity does not show differences since parecoxib is rapidly converted to valdecoxib, the pharmacologically active substance, by enzymatic hydrolysis in the liver, after that cytochrome P450 system metabolizes it to hydroxyvaldecoxib [16]. The same type of results were obtained with celecoxib, another COX-2 inhibitor, in Walker-256 tumor-bearing rats, where no differences in GST activity were observed [35]. The GSH:GSSG ratio, which lowers when oxidative stress increases, is mainly due to a decrease in the amount of GSSG (results not show), which may be related to the GR activity.

## 4. Materials and Methods 

### 4.1. Animals

The K14HPV16 strain was generously donated by Drs. Jeffrey Arbeit and Douglas Hanahan, from the University of California, through the USA National Cancer Institute Mouse Repository. Forty-three female, 20 weeks-old, FVB/n mice were used, including 21 wild-type (HPV16^−/−^) and 22 transgenic (hemizygotic HPV16^+/−^) animals. The animals were genotyped using previously described methods [36,37]. This study was approved by the University de Trás-os-Montes and Alto Douro Ethics Committee (approval no. 10/2013, approved on 8 July 2013) and the Portuguese Veterinary Directorate (approval no. 421/000/000/2014, approved on 24 September 2014).

### 4.2. Experimental Design

The animals were kept under controlled conditions such as temperature (23 ± 2 °C), relative humidity (50 ± 10%) and light-dark cycle (12h light/12h dark). Food and water were provided ad libitum and the health of animals was checked daily. The 43 animals were divided into four groups: group I (HPV16^−/−^, *n* = 10, treated with parecoxib); group II (HPV16^−/−^, *n* = 11, untreated); group III (HPV16^+/−^, *n* = 11, treated with parecoxib); group IV (HPV16^+/−^, *n* = 11, untreated). Parecoxib (5.0 mg/kg once daily) [31,32] was administered intraperitoneally for 22 consecutive days. Food and water consumption and body weight were registered weekly. All animals were euthanized 24 h after the last parecoxib administration using xylazine and ketamine followed by cardiac puncture exsanguination, as recommended by FELASA [38]. Skin samples (ear and chest skin), as well as internal organs (spleen, heart, liver, lungs, left kidney, right kidney and thymus) were collected. Liver samples were stored at −80 °C for posterior oxidative stress assessment.

### 4.3. HPV16-Induced Skin Lesions

The chest skin and ear samples were fixed in 10% neutral-buffered formalin, included in paraffin and stained with hematoxylin and eosin for histological analysis. Each sample was classified as normal skin, epidermal hyperplasia and epidermal dysplasia, as previously described [28]. Total infiltrating leukocytes and specific leukocytic populations (neutrophils, macrophages, lymphocytes, plasma cells, mast cells) were counted in high-power (400×) fields. Leukocyte counts were expressed as mean ± standard error to the mean (SEM).

### 4.4. Immunochemistry

The skin samples were tested immunohistochemically for expression of the proliferation marker ki67. Briefly, endogenous peroxidase was blocked using Refine Peroxide Block (Leica) and the slides were incubated with the primary antibody (D3B5, Cell Signaling, 1:1000) for 60 min. The refine (rabbit) polymer HRP (Leica) was used as secondary antibody and the antibody reaction was detected with 3.30-diaminobenzidine tetrachloride (DAB) for 10 min and counterstained with Mayer’s hematoxylin (4 min). Mouse spleen samples were used as positive controls and mouse IgG was used as an isotype control. Samples were classified as normal when ki67-positive cells were restricted to the basal layer of the epidermis or as pathological when suprabasal layers showed positive cells as well.

### 4.5. Humane Endpoints

Humane endpoints were assessed weekly by the same researcher, using a previously published scoring sheet [39]. Animals that reached a total score equal or greater than four at any time point were designated for euthanasia.

### 4.6. Biochemical Markers and Histology

Blood collected by cardiac puncture during euthanasia was stored in heparinized tubes, centrifuged at 1400× *g* for 15 min, and the plasma was separated and frozen at −80 °C for further analysis. Plasmatic concentrations of Alb, TP, glucose, ALT, AST and GGT were determined by spectrophotometric methods using an autoanalyzer (Prestige 24i, Cornay PZ). Histologically, liver samples were classified as normal liver or mild hepatitis, characterized by Kupfer cell hyperplasia with or without microabscesses.

### 4.7. Comet Assay

The alkaline comet assay was performed based on the method described by Collins [40], as adapted for high yield by Shaposhnikov et al. [41] and with an extra step of nucleoids digestion with formamidopyrimidine DNA glycosylase (Fpg), a DNA lesion-specific repair enzyme which converts oxidized purines into DNA single-strand breaks [42], generously donated by Professor Andrew Collins (University of Oslo, Norway). From liver samples, 0.2 g of liver were manually dissociated in Hank’s balanced salt solution at 4 °C to prepare a cell suspension. For preparing comet slides, 60 µL of cell suspension were mixed with 100 µL of 1% low melting point agarose, 6 µL of each sample were placed onto slides precoated with 1% normal melting point agarose. Each case was represented by two replicates. The slides were incubated at 4 °C for 5 min to solidify and immersed in a lysis solution (2.5 M NaCl, 0.1 M EDTA, 10 mM Tris, 1% Triton X-100, pH 10) at 4 °C, for 1 h. The slides were then washed (40 mM HEPES, 0.1 M KCl, 0.5 mM EDTA, 0.2 mg/mL bovine serum albumin, pH 8.0), three times, at 4 °C. Matched sets of slides were incubated with or without Fpg. For Fpg incubation, 10 µL of Fpg were applied to each mini-gel, and incubated at 37 °C for 30 min. Following incubation with or without the enzyme, slides were submitted to electrophoresis in 0.3 M NaOH and 1 mM EDTA for 30 min at 4 °C, 25 V and 300 mA (alkaline treatment). The slides were washed in PBS and distilled water at 4 °C, dehydrated in 70% and 96% ethanol for 15 min and air dried. The slides were then stained with 1 μg/mL of 4,6-diamidino-2-phenylindole (DAPI) solution (Sigma-Aldrich Chemical Company, Spain) and observed using an Olympus BX41 fluorescence microscope at 400×. The nucleoids were classified visually in five classes from 0 (no tail) to 4 (almost all DNA in the tail) (Collins, 2004). Fifty nucleoids were observed per mini-gel (100 per case). A genetic damage index (GDI), expressed in an arbitrary scale of 0 to 400, according to the formula:

GDI = (nucleoids class 0×0) + (nucleoids class 1×1) + (nucleoids class 2×2) + (nucleoids class 3×3) + (nucleoids class 4×4)

### 4.8. Oxidative Stress Parameters

Twenty-eight mice livers from the four experimental groups (*n* = 7) were used. The liver samples (≈ 0.25 g) were washed and added to 10% (p/v) phosphate buffer (KH_2_PO_4_, 50 mM, pH 7.0). After homogenization and sonication (six pulses of 20 s with 10 s intervals), the samples were subjected to three centrifugation cycles, at 4 °C: 800× *g* for 10 min, 10,000× *g* for 20 min and 12,000× *g* for 20 min. The supernatant obtained in the last centrifugation was used to measure antioxidant enzymes activities and GSH:GSSG ratio. The lipid peroxidation was determined using the pellets of second and third cycles. The total protein content of the supernatant and pellets obtained was evaluated by the Biuret method, using BSA as standard [43]. Total SOD activity method is based on inhibition of nitroblue tetrazolium (NBT) reduction by O2^−•^ generated by xanthine/xanthine oxidase system, according to the method originally described by Payá [44] with modifications [45]. To the 100 mM phosphate buffer, 10 mM NBT, 10 mM EDTA, 10 mM hypoxanthine and 23 mU/mol xanthine oxidase were added 10 µL of liver supernatant. The reduction of NBT was performed at 560 nm and 30 °C. One unit of SOD was defined as the enzyme amount causing 50% inhibition of NBT reduction rate and the result is expressed as U·min^−1^·mg protein^−1^. CAT activity was assayed polarographically using a Clark-type oxygen electrode as described before [46] in the presence of potassium phosphate buffer (50 mM, pH 7.0) and hydrogen peroxide (8.82 M). The reaction was carried out at 25 °C and is initiated by the addition of 10 µL of liver samples supernatant and the activity expressed in mmol H_2_O_2_·min^−1^·mg protein^−1^. GR activity was assayed as described previously [47]. The reaction system consisted of 100 mM phosphate buffer (pH 7.4), 0.5 mM EDTA, 1 mM oxidized glutathione (GSSG) and 0.1 mM NADPH. Enzyme activity was quantified at 25 °C by measuring the disappearance of NADPH at 340 nm and expressed as nmol NADPH oxidized. min^−1^·mg protein^−1^. GST activity was evaluated, as we described previously [48], by measuring the conjugation rate of 1-chloro-2,4-dinitrobenzene (CDNB) with GSH [49] at 340 nm (ε = 9600 M^−1^ cm^−1^) in 100 mM potassium phosphate buffer, 10% Triton X-100, 100 mM CDNB and 100 mM GSH. Lipid peroxidation was assayed by a modified TBA-based method for measuring the aldehydic lipid peroxidation decomposition derivatives (such as MDA), which form fluorescent products after reacting with TBA [50]. Then, 20 µL of pellet in 130 µL of buffer, 150 µL of TBA reagent and 2 µL of BHT 2% were incubated for 15 min at 100 °C. After cooling to room temperature were added 300 µL of butanol. After vortex and centrifugation (15,000× *g*, 3 min), the fluorescence of the upper butanol layer was measured at excitation 535 nm and emission 550 nm. The results are expressed as µmol MDA·mg protein^−1^. The GSH:GSSG ratio was calculated based on individual concentrations of glutathione reduced and oxidized that are determined spectrofluorimetrically using ortho-phthalaldehyde (OPA 1 mg/mL methanol) at excitation and emission wavelengths of 350 nm and 420 nm, respectively [51]. For GSH determination, samples were incubated in 100 mM potassium phosphate buffer (pH 8.0), 5 mM EDTA and 200 µL OPA at room temperature. For GSSG quantification, samples were first incubated for 30 min with 40 mM of *N*-ethylmaleimide (NEM) at room temperature and then incubated with 100 mM NaOH (pH 12.0) and 200 μL OPA to determinate GSSG content. Standard curves were prepared for calculation of GSH and GSSG concentrations.

### 4.9. Statistical Analysis 

Statistical analysis was performed with the IBM SPSS software (Statistical Package for the Social Sciences, Chicago, Illinois, EUA), version 25. A Chi-squared test was performed to study the distribution of histological lesions between groups. Student’s *t* tests were used to analyse results from the comet, the micronucleus and oxidative stress assays. For other variables, a two-way ANOVA followed by the Bonferroni test were performed. The results were expressed as mean ± standard deviation and values *p* < 0.05 were considered statistically significant.

## 5. Conclusions

The present results indicate that parecoxib is highly effective against HPV16-induced lesions, altogether abolishing the development of epidermal dysplasia. The mechanisms underlying its mechanism of action should be the focus of additional studies on this subject. Furthermore, parecoxib seems to attenuate oxidative stress induced by HPV-16 infection and showed a favorable toxicological profile, suggesting it is safe at the current dose in this model.

## Figures and Tables

**Table 1:** Relative organ weights (mean ± standard deviation).

Experimental Groups	Thymus	Heart	Lungs	Urinary Bladder	Spleen	Liver	Left Kidney	Right Kidney
Group I (WT + PX, *n* = 10)	0.0009 ± 0.0001	0.0041 ± 0.0002	0.0061 ± 0.0002	0.0009 ± 0.0002	0.0043 ± 0.0002	0.0520 ± 0.0018	0.0059 ± 0.0002	0.0059 ± 0.0002
Group II (WT untreated, *n* = 11)	0.0012 ± 0.0002	0.0042 ± 0.0002 ^a^	0.0063 ± 0.0003	0.0003 ± 0.0002	0.0047 ± 0.0002 ^a^	0.0574 ± 0.0012 ^a^	0.0057 ± 0.0002 ^a^	0.0062 ± 0.0002
Group III (HPV + PX, *n* = 11)	0.0009 ± 0.0001	0.0044 ± 0.0002	0.0054 ± 0.0001 ^a^	0.0009 ± 0.0002	0.0053 ± 0.0003 ^a^	0.0626 ± 0.0011 ^a^	0.0061 ± 0.0002 ^a^	0.0058 ± 0.0002 ^a^
Group IV (HPV untreated, *n* = 11)	0.0014 ± 0.0001	0.0051 ± 0.0002	0.0071 ± 0.0002	0.0008 ± 0.0001	0.0083 ± 0.0010	0.0717 ± 0.0019	0.0069 ± 0.0002	0.0068 ± 0.0002

^a^ Statistically significant difference from group IV (*p* < 0.05); HPV—human papillomavirus; PX—parecoxib; WT—wild-type.

**Table 2:** Incidence of skin lesions in each experimental group.

	Ear Skin Incidence/n (%)			Chest Skin Incidence/n (%)		
Experimental Groups	Normal	Hyperplasia	Dysplasia	Normal	Hyperplasia	Dysplasia
Group I (WT + PX, *n* = 10)	10/10 (100.0%)	0/10 (0%)	0/10 (0%)	10/10 (100.0%)	0/10 (0%)	0/10 (0%)
Group II (WT untreated, *n* = 11)	11/11 (100.0%)	0/10 (0%)	0/10 (0%)	11/11 (100.0%)	0/10 (0%)	0/10 (0%)
Group III (HPV + PX, *n* = 11)	0/10 (0%)	10/10 (100.0%)	0/10 (0%) ^a^	0/11 (0%)	10/10 (100.0%)	0/10 (0%) ^a^
Group IV (HPV untreated, *n* = 11)	0/10 (0%)	11/11 (100.0%)	7/11 (63.6%)	0/11 (0%)	11/11 (100.0%)	7/11 (63.6%)

^a^ Statistically different from group IV (*p* = 0.027). HPV—human papillomavirus; PX—parecoxib; WT—wild-type.

**Table 3:** Oxidative stress parameters.

Experimental Groups	CAT (mmol H_2_O_2_ min^−1^mg^−1^)	SOD (U min^−1^mg^−1^)	GST (mM CDNB min^−1^mg^−1^)	GR (µM NADPHox min^−1^mg^−1^)	GSH:GSSG	LPO (nM MDA mg^−1^)
Group I (WT + PX, *n* = 10)	0.026 ± 0.007	2.40 ± 0.24	0.73 ± 0.18	26 11 ± 2.90 ^a^	0.98 ± 0.14 ^a^	3.82 ± 1.94 ^a^
Group II (WT untreated, *n* = 11)	0.028 ± 0.003	2.77 ± 0.30	0.81 ± 0.30	20.67 ± 1.88	0.72 ± 0.12	10.39 ± 1.86
Group III (HPV + PX, *n* = 11)	0.022 ± 0.003	2.40 ± 0.41 ^b^	0.68 ± 0.22	23.82 ± 1.88	0.77 ± 0.14	7.96 ± 3.32
Group IV (HPV untreated, *n* = 11)	0.024 ± 0.001	3.30 ± 0.63	0.76 ± 0.22	23.50 ± 1.60	0.68 ± 0.11	12.07 ± 2.08

Values are the means ± SD of seven independent experiments performed in duplicate. HPV—human papillomavirus; PX—parecoxib; WT—wild-type. ^a^—Statistically significant differences in oxidative stress parameters versus group II (*p* < 0.05). ^b^—Statistically significant differences in oxidative stress parameters versus group IV (*p* < 0.05).

## Supplementary Materials

Supplementary materials can be found at https://www.mdpi.com/1422-0067/20/16/3902/s1.
