# Supplementary material for: The Cyclooxigenase-2 Inhibitor Parecoxib Prevents Epidermal Dysplasia in HPV16-Transgenic Mice: Efficacy and Safety Observations
Source: Int J Mol Sci. 2019 Aug 10;20(16):3902. doi: 10.3390/ijms20163902 (PMC6720853; doi:10.3390/ijms20163902)
Supplement: Supplementary file 1 [file ijms-20-03902-s001.pdf]

Supplementary table 1. Number and percentage of animals with change in body condition, coat and grooming, and ears, eyes and whiskers in different groups.

Group		Body Condition					Coat and grooming					Eyes, Ears and Whiskers				
			-1 Week	0 Week	1 Week	2 Week		-1 Week	0 Week	1 Week	2 Week		-1 Week	0 Week	1 Week	2 Week
I (*n* = 10)	*Wild-type* parecoxib	**0**	10 (100.0%)	10 (100.0%)	10 (100.0%)	10 (100.0%)	**0**	10 (100.0%)	10 (100.0%)	10 (100.0%)	10 (100.0%)	**0**	10 (100.0%)	10 (100.0%)	10 (100.0%)	10 (100.0%)
		**1**					**1**					**1**				
		**2**					**2**					**2**				
							**3**									
II (*n* = 11)	*Wild-type* control	**0**	11 (100.0%)	11 (100.0%)	11 (100.0%)	11 (100.0%)	**0**	11 (100.0%)	11 (100.0%)	11 (100.0%)	11 (100.0%)	**0**	11 (100.0%)	11 (100.0%)	11 (100.0%)	11 (100.0%)
		**1**					**1**					**1**				
		**2**					**2**					**2**				
							**3**									
III (*n* = 11)	HPV parecoxib	**0**	11 (100.0%)	8 (72.7%)	8 (72.7%)	7 (63.6%)	**0**	11 (100.0%)	11 (100.0%)	9 (81.8%)	10 (90.9%)	**0**	10 (90.9%)	10 (90.9%)	8 (72.7%)	7 (63.6%)
		**1**		3 (27.3%)	3 (27.3%)	4 (36.4%)	**1**			2 (18.2%)	1 (9.10%)	**1**	1 (9.10%)	1 (9.10%)	3 (27.3%)	4 (36.4%)
		**2**					**2**					**2**				
							**3**									
IV (*n* = 11)	HPV control	**0**	11 (100.0%)	10 (90.9%)	10 (90.9%)	10 (90.9%)	**0**	11 (100.0%)	11 (100.0%)	10 (90.9%)	8 (72.7%)	**0**	11 (100.0%)	11 (100.0%)	10 (90.9%)	11 (100.0%)
		**1**		1 (9.10%)	1 (9.10%)	1 (9.10%)	**1**			1 (9.10%)	3 (27.3%)	**1**			1 (9.10%)	
		**2**					**2**					**2**				
							**3**									
